# Climate change and children's health: resilience challenges for Brazil

**DOI:** 10.1016/j.jped.2024.11.002

**Published:** 2024-11-22

**Authors:** Mercedes Maria da Cunha Bustamante

**Affiliations:** Universidade de Brasília, Departamento de Ecologia, Brasília, DF, Brazil

**Keywords:** Extreme events, Adaptation, Health systems

## Abstract

**Objective:**

Three decades of evidence compiled by the Intergovernmental Panel on Climate Change (IPCC) reports is clear about the unequivocal impacts of humans on the global climate system are unequivocal and the wide range of effects on natural, social, and economic systems. Studies on impacts, vulnerability, and adaptation present the current impact on health and future consequences under different climate and greenhouse gas emissions scenarios. This article discusses some of the impacts of climate change on children's health which represents one of the most vulnerable groups.

**Sources:**

Evidence was sourced from recent scientific papers and reports referring to the potential impacts on children's health associated with the extreme events in Brazil observed in Brazil between 2023 and 2024 (heat waves and droughts, wildfires, and floods).

**Summary of the findings:**

Besides global warming, climate change is associated with more frequent and intense extreme events such as droughts, floods, and wildfires. Children and adolescents are particularly vulnerable due to physiological characteristics, interaction with exposure factors, and because they will live longer under changing conditions. Climate change projections and the intensification of impacts for Brazil highlight the adaptation challenges related to the protection of children under a changing climate and the role of the preparation of the country's health system, educators, and parents.

**Conclusions:**

The article underscores the need for collaboration among policymakers, health professionals, and educators, as well as the communities, to effectively address the adaptation challenges and build resilience to protect children against the impacts of climate change.

## Introduction

Almost 25 years ago, the Millennium Ecosystem Assessment, called by the United Nations and initiated in 2001, involved more than 1360 experts worldwide to assess the consequences of ecosystem change for human well-being. The reports also compiled actions that could enhance the conservation and sustainable use of ecosystems and their associated services.^1^ The report called the attention of the health sector to the complexities of the causal links between environmental change and human health because often, they are indirect, displaced in space and time, and dependent on several modifying forces.^2^ Health services and professionals should consider the diseases resulting from environmental degradation and the benefits that the natural environment provides to human health and well-being and their preservation for future generations.

The current concept of One Health considers health management in response to the rapidly accelerating environmental changes of the past century, recognizing that the health of humans, animals, plants, and the wider environment are closely linked and interdependent.^3^ However, despite the substantial body of evidence compiled by the Millennium Assessment and subsequent reports, environmental degradation is worsening in association with the global decline of biodiversity and climate change. The Earth's climate is changing alarmingly, with human-induced climate change already affecting many weather and climate extremes in all regions. In recent decades, a series of record-breaking events, such as storms, droughts, floods, and fires, underscores the urgent need for action.

Human influence has significantly warmed the atmosphere, the ocean, and the land. The scale of recent changes in the climate system as whole and many aspects of the climate system are unprecedented over many centuries to many thousands of years.^4^ The year 2023 was the warmest year since global records began in 1850 by a wide margin, with the temperature being 1.18 °C above the 20th century average of 13.9 °C and 1.35 °C above the pre-industrial average (1850–1900). The ten warmest years in the historical record have all occurred in the last decade (2014–2023), highlighting the gravity of the current situation.

Long-term global greenhouse gas emission scenarios compiled in the Intergovernmental Panel on Climate Change (IPCC) assessments enable analyses of future climate change, impacts, and response strategies (Lee et al. 2023). They indicate that global surface temperature will continue to rise until at least mid-century under all emissions scenarios considered. However, it is crucial to note that global warming of 1.5 °C and 2 °C will be exceeded during the 21st century unless deep reductions in carbon dioxide (CO_2_) and other greenhouse gas emissions occur in the coming decades, underscoring the importance of this action.

Human biology needs food, water, clean air, shelter, and relative climatic stability. Impacts on freshwater sources, food-producing systems, and climate regulation are related to major adverse health impacts. Direct and indirect mechanisms are associated with climate change's impacts on maternal and newborn health.^5^ Due to the long period over which the results of global warming will be felt, there is a disparity between the past generations responsible for emissions and the future ones who will experience their impacts.

By 2050, almost 70 % of the world's population will live in urban areas, many in unplanned or informal settlements. As a result, today's children and future generations are more likely to be exposed and vulnerable to climate change, related risks, and long-term effects on health and development.

Here, I discuss some of the impacts of climate change on children's health based on the evidence from recent literature but referring to extreme events in Brazil (heat waves and droughts, wildfires, and floods) observed between 2023 and 2024. I start by presenting information about climate change projections and impacts for Brazil and finalize by highlighting some considerations on adaptation challenges related to the protection of children under a changing climate in terms of food security and health system.

## Climate change impacts in Brazil: Current state and projections

The regional atlas published by the Intergovernmental Panel on Climate Change in the 6th Assessment Report,^6^ with a high confidence level, presents a consensus among scientists that for the Central and South America region, it is very likely that average temperatures have increased in all sub-regions and will continue to increase at rates higher than the global average, the South American monsoons (summer rains) will be delayed during the 21st century. There are projections of increased droughts and fire weather for the middle of the 21st century, considering 2 °C of global warming and above. The region is highly exposed, vulnerable, and heavily impacted by climate change. The impacts are exacerbated by poverty, population growth, high population density, loss of biodiversity, environmental degradation, and high dependence of national and local economies on natural resources to produce consumer goods.

A study about projections of extreme rainfall and hydro-geo-meteorological disaster risks in Brazil considering global warming scenarios of 1.5, 2.0, and 4.0 °C pointed out a significant change in intense rainfall events and increasing risks of landslides and flash floods.^7^ South and Southeast regions are particularly vulnerable and also concentrate densely populated areas.

Although the warming of South America closely follows the global path, the rise of temperatures has been more pronounced in some regions, which have also seen a parallel increment in the occurrence of droughts and fire weather.^8^ Reanalysis datasets since 1971, the frequency of these compound extremes has surged in critical South American regions, including the northern Amazon, which has seen a 3-fold increase in the number of days per year with extreme fire weather conditions (including high temperatures, dryness, and low humidity). Also, the surface temperature of the tropical Pacific Ocean modulates the interannual variability of dry compounds in South America.

The Brazilian population has been strongly affected by extreme climate events in recent years. The indicator of the Brazilian Ministry of National Integration (Integrated Disaster Information System - S2ID) regarding the risk of death, number of deaths, missing persons, and directly affected persons attributed to disasters per 100,000 inhabitants between 2015 and 2021, Brazil shows significant differences between the Brazilian states from year to year. While some states record more than 5700 deaths, disappearances, or people affected per 100,000, others have rates close to zero. Despite the variations, there is an upward trend in the number of victims of catastrophic events, and 2020 and 2021 showed a significant increase in the indicator. In 2021, there were 1032 victims per 100,000 inhabitants - or more than 2 million people. Estimates for 18 Brazilian state capitals, in at least 10, between 1997 and 2011, more than 800 deaths in these cities occurred only due to the increase in temperatures caused by climate change.^9^ In 2024, after unprecedented flooding in the federal of Rio Grande do Sul, over 60 million people in Brazil are facing higher temperatures during the winter months (August-September 2024), while devastating wildfires have been impacting four biomes (Amazonia, Pantanal, Cerrado, and Atlantic Forest). The transportation of smoke from wildfires affected human health and disrupted daily life in many parts of Brazil. Also, the heat experienced by Brazilians during the winter and spring of 2024 has a clear connection to climate change, with forecasted high temperatures made more likely due to global warming.

## Climate change - Children at risk

The Children's Climate Risk Index (CCRI)^10^ comprehensively analyzes climate risk from a child's perspective. It ranks countries based on children's exposure to climatic and environmental shocks, such as cyclones and heat waves, and their vulnerability to these shocks based on access to essential services. Brazil is considered a high-risk country for children and adolescents exposed to climatic and environmental shocks. Only Mexico has a higher index in Latin America and the Caribbean. In the case of Brazil, more than 40 million children and adolescents are exposed to more than one of the risks analyzed in the study, representing almost 60 % of children and adolescents in the country. For example, more than 8.6 million Brazilian boys and girls are exposed to the risk of water shortages; and more than 7.3 million are exposed to the risks of river flooding.

In another document,^11^ UNICEF stressed the urgency of discussing climate change in Brazil, focusing on children and adolescents as they are not only at a more sensitive stage of development but also suffer the most from the impacts. Climate change and environmental degradation also jeopardize services, policies, and institutions that meet the needs of children and their families.

Also, changing climate patterns have direct and indirect effects on pregnant women affecting maternal and newborn health, with more severe impacts in climate-vulnerable regions where access to resources is limited. Increased pregnancy loss, premature birth, serious maternal illnesses, and cognitive impacts on offspring are some of the risks.

## Heat-related illnesses

Global projections indicate that the population exposed to deadly heat stress can increase. By the end of the century, high climate change and population growth scenarios showed approximately 5-, 10-, and 100–1000-fold increments in the population exposed to a mean hottest monthly temperature of 30 °C, 35 °C, and 40 °C, respectively.^12^ With global warming higher than 4 °C by 2100, the number of days with stressful weather conditions for outdoor workers will increase by up to 250 working days per year by the end of the century in parts of Central and South America (https://www.ipcc.ch/report/ar6/wg2/about/frequently-asked-questions/).

Thermal limits become critical with more frequent, intense, and longer-lasting heat waves due to climate change. Wet-bulb temperature (WBT) combines dry air temperature (as can be seen on a thermometer) with humidity, a measure of human heat-stress conditions. The amount of water vapor in the atmosphere grows at roughly 6 to 7 % per degree of warming (Clausius–Clapeyron effect). Previous evidence suggested a wet-bulb temperature (Tw) of 35 °C as a theoretical upper limit on human abilities to thermoregulate biologically.

However, the use of a more accurate threshold and the latest coupled climate models quantified the exposure to potentially lethal heat for future climates with different global warming levels.^13^ The study indicated that humans are more vulnerable to moist heat stress than previously considered because of lower thermal limits. In the future, moist heat extremes will be outside the range of past human experience meaning that current heat mitigation strategies for billions of people will not be effective. While some physiological adaptation from the new thresholds is possible, additional behavioral, cultural, and technical adaptative measures will be required to maintain healthy lifestyles. However, most of all, keeping warming under 2 °C nearly eliminates exposure and risk of widespread moist heatwaves while a sharp rise in exposure occurs at 3 °C of warming.

According to the National Institute of Meteorology (INMET) and considering the historical series, 2023 was the hottest year in Brazil, with a mean temperature of 24.92oC (INMET). A heatwave is characterized by an increase in temperatures above the average of 5 °C for five days or more. In 2023, the country faced nine episodes of heat waves, partially due to the El Nino, which tends to favor higher temperatures. In 2024, until the end of September, Brazil experienced eight heat waves.

Evidence showed that child-adult differences in thermoregulation are less evident during mild and moderate heat exposure, but heat illness is enhanced at environmental extremes, placing children at increased risk.^14^ Adverse effects on the thermoregulatory physiology of children can be exacerbated by other stressors such as pollution, ultraviolet radiation, obesity, diabetes, associated comorbidities, and polypharmacy that are more commonly occurring at younger ages. An experiment with 34 young children (aged 6 months–8 years, boys and girls) showed the development of thermoregulatory responses with growth and that there is a disadvantage by immature mechanisms and small body size in younger children.^15^

## Air quality - Wildfire smoke

In the second half of July 2024, the European Atmosphere Monitoring System (Copernicus) reported a substantial increase in fires and associated emissions across South America, with most wildfire activity noted in parts of the Brazilian Amazon and Bolivia. In Brazil, total cumulative emissions until September 2024 were higher than average at around 180 megatonnes of carbon and followed the record emissions year 2007 trajectory. Emissions in September so far had accounted for almost 60 megatonnes of this total. The smoke transport impacted air quality in at least eight federal states and the Federal District.

The combination of higher temperatures and lower air humidity increases the risks of air pollution, particularly those associated with emissions from wildfires. Children are especially vulnerable to air pollution because they develop rapidly, particularly their immune systems and lungs (https://ceh.unicef.org/spotlight-risk/wildfire-smoke).^16^,^17^ Also, they breathe more rapidly than adults, take in more air relative to their body weight, and have less nasal deposition of particles, meaning that a higher proportion of particles can penetrate deeply into the lungs.

As mentioned before, climate change is associated with creating conditions such as increased drought, longer and more intense heatwaves, low relative humidity, dry lightning, and strong winds, all contributing to hotter, drier, and longer fire seasons. Wildfires are projected to become more frequent and intense, with a global increase of extreme fires from current levels to 14 % more by 2030, 30 % more by the end of 2050, and 50 % more by the end of the century.^18^

Wildfire smoke is an increasing public health concern. Recently, a systematic review of epidemiological studies analyzed the association between wildfire smoke and the health of children and adolescents.^19^ Most studies found that wildfire smoke was associated with multiple adverse health outcomes, such as respiratory morbidities, among children and adolescents. Smoke from forest fires contains particles of 2.5 micrometers (PM2.5) in diameter or smaller. Exposure to particulate matter from fire increases child mortality. Each 1 μg/m3 increment of PM2.5 from fires was associated with a 2.3 % increase in the risk of child mortality. PM2.5 released from wildfires is approximately 10 times more harmful to respiratory health than PM2.5 from other sources, particularly in the vulnerable age group of 0 to 5 years. Global mortality related to wildfire smoke was estimated to be 677,745 deaths annually, with approximately 40 % occurring in children younger than 5 years of age. In-utero exposure to wildfire smoke may increase the risk of adverse birth outcomes and have long-term impacts, elevating the risks of preterm birth and low birth weight.

## Floods

In 2024, the Brazilian federal government mapped 1942 municipalities susceptible to disasters associated with landslides, flooding, and flash floods, which represents almost 35 % of all Brazilian municipalities (https://www.sgb.gov.br/prevencao-de-desastres). The areas within these 1942 cities are home to more than 8.9 million Brazilians, representing 6 % of the national population. Compared with the mapping conducted in 2012, the new survey added new criteria and databases, and the number of municipalities considered susceptible to disasters increased by 136 %. With the data until 2022, the states with the highest proportion of the population in risk areas are Bahia (17.3 %), Espírito Santo (13.8 %), Pernambuco (11.6 %), Minas Gerais (10.6 %) and Acre (9.7 %). The states with the most protected population against disasters are the Federal District (0.1 %), Goiás (0.2 %), Mato Grosso (0.3 %) and Paraná (1 %).

A global review of water-related disasters and their health impacts^20^ pointed to short-term health impacts due to water-borne and vector-borne diseases and physical health problems such as injuries. Long-term health impacts include mental health problems and malnutrition.

The risk of infectious disease outbreaks was particularly worrisome^21^ in the case of the floods that devastated Rio Grande do Sul State in Brazil in April 2024 and affected approximately 90 % of the municipalities and over 2 million people.

Women and children are more vulnerable than other groups.^22^,^23^ Among the children, those under the age of 5 are more affected by water-borne diseases, malaria, and wasting in a disaster situation. Also, there are indications of adverse effects of indoor mold and irritants on children, and they are more dependent on adults to be safe and maintain hygienic conditions.

Short-lived or protracted displacement can multiply climate-related risks for children and their families.^24^,^25^ After a disaster, children may become separated from their parents or caregivers, amplifying the risks of exploitation, child trafficking, and abuse, disrupting access to education and healthcare, and exposing children to malnutrition, disease, and inadequate immunization, with physical and mental health consequences.

## Food and nutritional security (FNS)

According to the World Bank, global food insecurity has affected 135 million in 53 countries in 2019 and increase significantly to 345 million in 82 countries in 2022. (https://www.worldbank.org/en/news/feature/2022/10/17/what-you-need-to-know-about-food-security-and-climate-change/). Climate change has direct and significant impacts on food insecurity. As global temperatures rise, food production becomes more complex and uncertain due to changing weather patterns, extreme weather events, and other environmental disruptions. As global temperatures rise, food production becomes more complex and uncertain due to changing weather patterns, extreme weather events, and other environmental disruptions. These challenges have far-reaching implications for food supplies around the world. Climate change risks to FNS interact strongly with other social and economic risks and lead to compound or cascading risks.

The risks associated with FNS, and climate change are expressed by the number of people at risk of hunger and malnutrition. Climate change is expected to affect these risks to FNS through falls in agricultural productivity, reduced income, emerging food security problems and disruptions in food distribution, lower nutrient content of some crops, and changes in diet quality.^26^

The link between climate change and human nutrition goes beyond questions of calorie availability.^27^ The provision of nutritious and affordable diets will represent a growing challenge by 2050 as more severe and extreme events increase the risk of acute food insecurity and malnutrition. Climate change will affect many determinants of micronutrient deficiency, especially the availability of and access to fruit and vegetables. Higher concentrations of atmospheric CO_2_ are linked to reducing the protein and mineral content of cereals, reducing food quality.^28^,^29^

Adverse effects related to malnutrition and stunting, diet-related child morbidity and mortality, and increased disability-adjusted life years lost. Stunting in early childhood will have lifelong health implications, with intergenerational transmission of the effects of malnutrition. For example, stunting in mothers is associated with low birth weight in their children.^26^

## Role of parents and educators in creating safe spaces

Climate change education is about learning in the face of risk, uncertainty, and rapid change.^30^ The education sector can play an essential role in adaptation.^31^ A robust and rapidly growing academic literature is documenting the negative consequences of climate change, particularly high temperatures for students.^32^,^33^ This link is important because projections show that much of the world will be exposed to substantially higher temperatures in a warmer world. Integrating climate change education into all formal education systems is not just important; it's crucial. Thanks to the multiplier effect that benefits families and communities, it's one of the most effective means of developing capacities to deal with the climate crisis.

As children are particularly vulnerable to the effects of extreme events, school curricula that incorporate learning about local disaster risks and how to prepare for and deal with them when they occur can increase young people's resilience. Community-based, child-centered disaster risk reduction will increasingly become part of teachers' work. Research and education policy play key roles in finding ways to adapt schools and ways of educating students to reduce the negative consequences of exposure to climate extremes. Promoting climate adaptation for young students may be the most consequential way for the education sector to engage with the new challenges posed by climate change.

The collaboration between families and educational and health systems can also reinforce the relevance of healthy lifestyle choices through the provision of balanced diets with locally sourced, quality foods, when possible, to reduce carbon footprints and encourage outdoor play and activities to promote physical health and well-being. Finally, parents and educators are also critical actors in addressing children's concerns, listening to their fears and anxieties about climate change, providing reassurance and guidance, and fostering resilience by teaching coping strategies to help children deal with uncertainty and stress related to environmental issues and skills that help children adapt to change and develop a sense of agency.

## Responses of health systems: building climate-resilient systems

Increased frequency, intensity, and duration of extreme events affect large areas and an increasing share of the Brazilian population. Effective preparation and response will demand adequate training, transdisciplinary work, robust finance, and efficient coordination between municipal, state, and national health systems and governance. Some events, such as extensive wildfires, generate transboundary effects that imply international articulation. The preparation should include considerations before, during, and after the events to optimize public health outcomes in the short and long term.^34^

Experience exchange is critical to evaluating commonalities of existing climate-resilient health care and disaster management frameworks in different contexts. The World Health Organization^35^ designed an Operational Framework for climate-resilient and low-carbon health systems to assist decision-makers in health systems, including public health agencies, with comprehensive planning involving factors like financing, governance, health workforce, information systems, infrastructure, supply chains, technologies, and community interactions.

It is crucial to consider the disparities in impact with vulnerable groups such as children and adolescents, particularly those from low-income communities, and Indigenous Peoples, and disproportionately affected, that face unique challenges.

## Conclusions

As presented by the IPCC's 6th Assessment Report,^36^ climate-resilient development combines climate change adaptation strategies with actions to reduce greenhouse gas emissions to support sustainable development for all. However, if temperatures exceed 2 °C of warming by 2100, climate-resilient development will become impossible in some regions.

Adaptation is essential to reducing damage, but to be effective, ambitious reductions in greenhouse gas emissions must accompany it. As warming increases, the effectiveness of many adaptation options decreases. Also, progress on adaptation still needs to be improved, with large gaps between adaptation actions and the demands in many regions.

The constraints faced by the natural world and people, especially at higher degrees of warming, limit the availability of adaptation options. Different barriers (biophysical, institutional, financial, social, and cultural) can result in soft and hard adaptation limits, especially when combined.

The adverse effects of climate change on children are multifaceted and highly dependent on age, education, social and economic contexts. Governments must integrate climate considerations into health policy and planning. Health systems and professionals should prepare facilities to enhance readiness, collaborate with communities, and advise on the physical and mental health risks of children and adolescents, protecting them from climate change and displacement impacts.

Both parents and educators, at all levels, play crucial roles in mitigating the health impacts of climate change on this vulnerable group. By fostering awareness, promoting healthy lifestyles, engaging in community efforts, and preparing children and young people to live in a climate-changed world, they can help build a healthier and more resilient future for the next generation.

## Funding sources

Rede Clima (MCTI), INCT Mudança do Clima (CNPq, FAPESP, CAPES).

## Conflicts of interest

The authors declare no conflicts of interest.
